# Supplementary material for: Balancing fire risk and human thermal comfort in fire-prone urban landscapes
Source: PLoS One. 2019 Dec 27;14(12):e0225981. doi: 10.1371/journal.pone.0225981 (PMC6934286; doi:10.1371/journal.pone.0225981)
Supplement: S1 File — (PDF) [file pone.0225981.s001.pdf]

S1. Forest Flammability Model inputs.

S1 Table A. Description of plant traits used in the FFM for this study.

Species	Plant Traits											
	Leaf form	Thickness (mm)	Thickness range	Width (mm)	Width range	Length (mm)	Length range	Separation	Branch orders	Branch order range	Clump separation	Clump diameter
Quercus robur	Flat	0.18[1]	0.15	60[1]	80	104.3[1]	6	0.92	5.7	3	0.367	2.43
Acacia implexa	Flat	0.34[2]	0.09	1.16[2]	0.2	129.4[2]	19	1.168	5.2	1	0.153	0.882
Tristaniopsis laurina	Flat	0.36		25.6		130		0.945	4.87	1	0	3.17
Eucalyptus camaldulensis	Flat	0.3[3]	0.1	24.2[3]	28	106.9[3]	94	1.9	4.1[3]	4[3]	1.3[3]	1.4[3]
Eucalyptus sideroxylon	Flat	0.36[2]		18.3[2,4]	6	103.2[2,4]	70	1.43	4.51[3]	2.73[3]	0.9[3]	1.18[3]
Bursaria spinosa	Flat[5]	0.24[5]	0.06	4.3[5]	3	14.8[5]	11	0.25[5]	4.5[5]	0	0.1	0.9
Poa labillardieri	Flat	0.25[5]	0.22	1.3[5]	0.5	150[5]	0	0.92[5]	1[5]	0	0	1

All data collected for this study except as indicated by the following superscripts;

1. Wirth C, Lichstein JW. The Imprint of Species Turnover on Old-Growth Forest Carbon Balances - Insights From a Trait-Based Model of Forest Dynamics. . In: Wirth C, Gleixner G, Heimann M, editors. Old-Growth Forests: Function, Fate and Value. New York, Berlin, Heidelberg.: Springer; 2009. p. 81-113.
2. Gill, A.M.; Moore, P.H.R. Some ecological research perspectives on the disastrous Sydney fires of January 1994. In: Proceedings of the 2nd International Conference on Forest Fire Research Volume I: pp. 63-72; 1994
3. Collins, L., Penman, T. D., Price, O. F., & Bradstock, R. A. (2015). Adding fuel to the fire? Revegetation influences wildfire size and intensity. *Journal of environmental management*, *150*, 196-205.
4. Boland DJ , Brooker MIH , Chippendale GM , Hall N , Hyland BPM , Johnston RD , Kleinig DA , McDonald MW , Turner JD (2006) 'Forest trees of Australia.' 5th edn. (CSIRO Publishing: Melbourne)
5. Zylstra P BR, Bedward M, Penman TD, Doherty MD, Weber RO, et al. . Biophysical Mechanistic Modelling Quantifies the Effects of Plant Traits on Fire Severity: Species, Not Surface Fuel Loads, Determine Flame Dimensions in Eucalypt Forests. PLoS ONE. 2016;11(8): e0160715.

S1 Table B. Species size description and inputs.

Species Arrangement	propDEAD	heME	heRa	htME	htRa	hcME	hcRa	hpME	hpRa	wME	wRa
Quercus robur	0	4.4	2.35	9.47	6.625	1.87	1.9	12.01	9	18.69	8
Acacia implexa	0	3.29	1.45	4.61	2.68	1.525	1.7	5.7	3.5	4.89	2.25

Tristaniopsis laurina	0	2.41	2.175	3.94	1.825	1.79	1.6	4.56	2.4	3.145	1.95
Eucalyptus sideroxylon	0	4.26	5	9.386	4.6	1.591	3.4	12.055	6.2	10.782	12.9
Eucalyptus camaldulensis	0	7.53	11.325	14.38	10.87	4.494	8.2	17.424	14	18.456	15.8
Eucalyptus camaldulensis Clumped	0	5.61	5.08	10.9	4.08	2.97	6.2	13.55	5.2	15.28	6.8
Eucalyptus camaldulensis Dispersed	0	9.22	4.33	17.36	6.98	5.15	6.7	21.43	8.9	20.65	5.05
Eucalyptus camaldulensis Continuous	0	8.47	3.18	14.33	7.03	5.54	3.7	17.26	9	19.63	15.8
Bursaria spinosa	0	0.114	0.55	3.26	2.6	0.114	0.55	3.264	2.6	4.94	4
Poa labililardieri	0	0	0			0	0	0.838	0.5	0.794	0.5

S1 Table legend.

**Symbol**	**Description**	**Unit**
H_c	Height to the bottom centre of a plant crown	cm
H_e	Height of the bottom edge of a plant crown	cm
H_p	Height of the top centre of a plant crown	cm
H_t	Height of the top edge of a plant crown	cm
propDEAD	Percentage of plant dead	%
Ra	Range	
W	width	
ME	mean	

S1 Table C. Plant spacing inputs.

**Planting Arrangement**	**Code**	**Species**	**Plant separation mean (m)**	**Plant separation Range (m)**
Clumped	CQR	*Quercus robur*	5	6
Dispersed	DQR	*Quercus robur*	50	30
Continuous	XQR	*Quercus robur*	15	10
Clumped	CAI	*Acacia implexa*	2.25	3.2
Dispersed	DAI	*Acacia implexa*	12.4	10.1
Continuous	XAI	*Acacia implexa*	5.9	3.15
Clumped	CTL	*Tristaniopsis laurina*	1.5	2
Dispersed	DTL	*Tristaniopsis laurina*	9.5	6
Continuous	XTL	*Tristaniopsis laurina*	6	10

Clumped	CES	*Eucalyptus sideroxylon*	4	1.1
Dispersed	DES	*Eucalyptus sideroxylon*	25.7	16.9
Continuous	XES	*Eucalyptus sideroxylon*	9.55	5.6
Clumped	CEC	*Eucalyptus camaldulensis*	3.5	4
Dispersed	DEC	*Eucalyptus camaldulensis*	36.4	35
Continuous	XEC	*Eucalyptus camaldulensis*	7.9	12.5
Elevated fuels		*Bursaria spinosa*	1.73	1.09
Near surface fuel		*Poa labillardieri*	0.47	0.19
