# Supplementary material for: Balancing fire risk and human thermal comfort in fire-prone urban landscapes
Source: PLoS One. 2019 Dec 27;14(12):e0225981. doi: 10.1371/journal.pone.0225981 (PMC6934286; doi:10.1371/journal.pone.0225981)
Supplement: S2 File — (PDF) [file pone.0225981.s002.pdf]

S2. Rayman Model Inputs.

S2 Table A. Photo plots for hemispherical imagery

Planting	Canopy Species	Mean Plant Separation (m)	Mean Canopy diameter (m)	Min. No photos per plot	Plot Size (m^2)	Location/s
Clumped	*Quercus robur*	5	18.69	10	25	Fairfield Park;<br>Merri Creek
Dispersed	*Quercus robur*	50	18.69	10	25	Johnson Park Northcote; Campbells Croft Vermont;<br>Yarra Bend Park
Continuous	*Quercus robur*	15	18.69	10	25	Apex Park Creswick
Clumped	*Acacia implexa*	2.25	4.74	10	10	Darebin Parklands;<br>Goulburn River Walk Seymour
Dispersed	*Acacia implexa*	12.4	4.74	10	10	Yarra Bend Park; street trees in Wallan, Brunswick and Northcote
Continuous	*Acacia implexa*	5.9	4.74	10	10	All Nations Reserve Northcote
Clumped	*Tristaniopsis laurina*	1.5	3.145	10	10	Dixon Street Northcote
Dispersed	*Tristaniopsis laurina*	9.5	3.145	10	10	Beavers Street Northcote
Continuous	*Tristaniopsis laurina*	6	3.145	10	10	O'Gradys Street Clifton Hill
Clumped	*Eucalyptus sideroxylon*	4	10.78	10	20	All Nations Reserve
Dispersed	*Eucalyptus sideroxylon*	25.7	10.78	10	20	Yarra Bend Park
Continuous	*Eucalyptus sideroxylon*	9.55	10.78	10	20	Fairfield Park
Clumped	*Eucalyptus camaldulensis*	3.5	15.28	10	25	Darebin Parklands
Dispersed	*Eucalyptus camaldulensis*	36.4	20.65	10	25	Kings Park Seymour; Yarra Bend Park
Continuous	*Eucalyptus camaldulensis*	7.9	19.63	10	25	Broadford Common

S2 Table B. Personal data inputs for the RayMan model to enable PET to be examined.

**Personal Data**	
Height	1.75m
Weight	75kg
Age	35
Sex	Male
Activity	80W
Clothing	0.90 clo
